# Identification and Functional Analysis of Genome Mutations in a Fluoride-Resistant *Streptococcus mutans* Strain

**DOI:** 10.1371/journal.pone.0122630

**Published:** 2015-04-09

**Authors:** Ying Liao, Jianwei Chen, Bernd Willem Brandt, Yuanfang Zhu, Jiyao Li, Cor van Loveren, Dong Mei Deng

**Affiliations:** 1 State Key Laboratory of Oral Diseases, West China Hospital of Stomatology, Sichuan University, Chengdu, China; 2 Department of Preventive Dentistry, Academic Centre for Dentistry Amsterdam, University of Amsterdam and VU University Amsterdam, Amsterdam, The Netherlands; 3 BGI-Shenzhen, Shenzhen, China; Oregon Health and Science University, UNITED STATES

## Abstract

It is known that fluoride-resistant microorganisms are different from fluoride-sensitive ones in growth, adherence and metabolic activity. It was hypothesized that these phenotypic differences were due to stable genotypic changes in the fluoride-resistant strains. However, until now, no studies have reported these genotypic changes. The aim of this study is to identify such changes in a fluoride-resistant *Streptococcus mutans* strain (C180-2FR) using whole-genome shotgun (WGS) sequencing and to examine the potential function of the identified mutations by comparing gene expression between the fluoride-sensitive (C180-2) and C180-2FR strains. We performed 50 bp paired-end Illumina shotgun sequencing for both strains. Through extensive bioinformatic analysis, we were able to identify 8 single nucleotide polymorphisms (SNPs) in the genome of C180-2FR, which were further confirmed by Sanger sequencing. Expression of the genes containing or in proximity to the SNPs in C180-2 and C180-2FR was then quantified by real-time PCR. A gene cluster containing genes coding for fluoride antiporters was up-regulated 10-fold in C180-2FR when compared to that in C180-2, independent of growth phase. Two SNPs are located in this gene cluster, one in its promoter region and the other in its protein-coding region. In addition, one gene, which codes for a putative glycerol uptake facilitator protein, was found to be down-regulated by 60% in C180-2FR at an early growth phase. The promoter region of this gene contained a SNP. No difference in expression was found for the other SNP-containing genes. In summary, using WGS sequencing, we were able to uncover genetic changes in the genome of a fluoride-resistant strain. These findings can provide new insights into the mechanism of microbial fluoride resistance.

## Introduction

Fluoride is still the most effective caries-preventive agent [1]. Since its discovery as a preventive agent in 1931, fluoride has been widely used in many caries-preventive products and in many countries [2,3]. While the application of fluoride containing products has markedly reduced caries [4], the exact mechanism of fluoride in caries prevention is still not completely understood. It is known that fluoride prevents caries through a dual-mode of action; it not only inhibits the demineralization and enhances the remineralization of dental hard tissues but also affects bacterial growth and microbial metabolic activity by the inhibition of enolase and ATPase [5].

One of the consequences of the widespread and prolonged application of fluoride is the risk of the development of fluoride resistance in microorganisms. Fluoride-resistant strains have been discovered in several clinical studies [6,7], where fluoride-resistant *Streptococcus mutans* colonies were recovered from xerostomia patients who had been treated with gels containing a high concentration of NaF. To better understand the mechanism of microbial fluoride resistance, researchers also created fluoride-resistant strains in the laboratory by selecting colonies that were able to grow in the presence of 400–600 ppm fluoride. To date, fluoride-resistant strains have been created for several streptococci, including *S*. *mutans*, *S*. *sobrinus* and *S*. *salivarius* [8–10]. These fluoride-resistant strains demonstrated clear phenotypic differences in growth, adherence and metabolic activity compared to the fluoride-sensitive strains [6,10,11]. However, there is debate over whether these phenotypic differences were due to a stable genotypic resistance or due to a temporary adaptation [8,12–14]. The evidence collected so far seems to support the former. For example, fluoride resistance could be maintained after the microorganisms were cultured in fluoride-free growth medium for 20–30 generations or sometimes for more than 500 generations [6,8]; the fluoride-sensitive strain could become fluoride resistant upon transformation with genomic DNA isolated from the fluoride-resistant strain [15]. Brussock *et al*. [16] proposed that the high levels of fluoride resistance might be due to a cumulative effect of mutations in at least two genes.

Despite ample indirect proof, no studies have yet been able to identify genotypic changes in the fluoride-resistant strains [17,18]. A candidate gene approach was usually employed in these studies. With this approach, candidate genes were selected for sequencing based on expert knowledge. For example, we sequenced two glycolytic enzymes, enolase and ATPase, obtained from fluoride-sensitive and fluoride-resistant strains [17] because it is known that their function can be affected by fluoride [7,19]. However, the sequences of both enzymes were identical in both strains ([17] and our unpublished data). In another study, the *fabM* gene, a regulator for the synthesis of monounsaturated fatty acids, was significantly up-regulated in a fluoride-resistant strain relative to a fluoride-sensitive strain. This gene was also selected for sequencing, but no mutation was found [18]. Taken together, the candidate gene approach is not ideal for the identification of genotypic changes in fluoride-resistant strains. Alternatively, one could sequence the whole genome of both strains and make a comparison. However, this method is still time consuming and costly.

A recently developed state-of-the-art molecular technique, whole-genome shotgun (WGS) sequencing, appears to be a good alternative. High-throughput sequencing, followed by extensive bioinformatics analysis, can reveal a tremendous amount of information about the target organisms. This technique has been successfully applied to predict single-nucleotide polymorphisms (SNPs) associated with bacterial antibiotic resistance and host adaptation [20,21].

The aims of our study are to identify genotypic changes in a fluoride-resistant *S*. *mutans* strain using the WGS technique and to examine the potential function of the identified mutations. To this end, the genomes of the fluoride-sensitive and fluoride-resistant *S*. *mutans* strains were WGS sequenced and analyzed. Next, the identified mutations were confirmed by traditional Sanger sequencing. The expression of the genes containing the confirmed mutations was further compared between these two strains.

## Materials and Methods

### Bacterial strains and growth conditions

The strains used in this study were *S*. *mutans* C180-2 [22] and the derived fluoride-resistant strain C180-2FR [7]. These two strains were chosen because the process of obtaining a C180-2 fluoride-resistant strain in the laboratory is well documented [23]. The phenotypic differences between the two strains have been described previously [6,19]. Both strains were grown in Brain Heart Infusion (BHI) broth or on BHI agar anaerobically (90% N_2_, 5% CO_2_, 5% H_2_) at 37°C. The strains were also grown on TYCSB agar [24] when indicated. The characteristic components of TYCSB agar are 20% sucrose and 0.1 unit/ml bacitracin.

### Characterization of *S*. *mutans* C180-2 and C180-2FR

*S*. *mutans* C180-2 and C180-2FR were characterized by examining growth in BHI broth (without the addition of fluoride) and fluoride resistance. To test the growth of *S*. *mutans*, an overnight culture of each strain was diluted 20 times in fresh BHI broth. The optical density at 600 nm (OD_600_) of the culture was measured at various time points until the bacterial cells reached stationary phase. To test the resistance to fluoride, *S*. *mutans* C180-2 and C180-2FR cultures (approximately 5×10^8^ CFU) were plated on BHI agar plates containing either no fluoride or 80–500 ppm fluoride and incubated anaerobically at 37°C for 3 days. The presence of colonies was used to indicate the growth of either strain.

### Genomic DNA extraction and whole-genome shotgun (WGS) sequencing

Once the fluoride resistance of *S*. *mutans* C180-2FR was confirmed, genomic DNA was extracted from the culture of *S*. *mutans* C180-2 or C180-2FR using the Thermo Genejet Genomic DNA purification kit (Thermo Scientific, MA, USA). The genomic DNA was sequenced on an Illumina GAIIx platform (50 bp paired-end reads: mean insert size 250 bp) by BaseClear B.V. (Leiden, the Netherlands).

The sequencing data were further processed and assembled as follows: the reads were first filtered on length (minimum: 50 bp). Next, the reads were filtered on quality: low quality reads (20 consecutive bases Q ≤ 20) were removed, as well as reads with ≥10% ambiguous (N) bases. In addition, duplicate reads were discarded. Finally, the assembly was conducted using SOAP_denovo 2.04 [25]. Several values for K-mer length were used to optimize the assembly. The values that resulted in the best N50 contig length were chosen: 39 for *S*. *mutans* C180-2 and 37 for C180-2FR.

### Small size insertion-deletion variants calling

After mapping the quality-filtered short reads of C180-2 to those of C180-2FR with BWA 0.58 [26], we used Dindel 1.01 [27] with standard parameters to identify the potential insertions and deletions (InDels). InDels with a length less than 10 bp were selected. From this selection, InDels with more than one mismatch in the region 10 bp up- and down-stream or with one or more gaps in the region 25 bp up- and down-stream were eliminated because these types of InDels were most likely due to erroneous mapping. The predicted InDels were further validated using the quality-filtered short-read data: InDels covered by less than three reads were removed, and InDels located in repeat regions were filtered out to avoid ambiguous mapping.

### SNPs calling

MUMmer3.22 [28] was used to align the genome sequence of the reference strain *S*. *mutans* LJ23 (NC_017768) to the tested strains and to list all homozygous SNPs (with default parameters). To improve the sensitivity and accuracy of SNP detection, the quality-filtered short reads were mapped to these SNP areas with SOAPaligner/soap2 [29]. SNPs that mapped to a gap, to repeat regions or near a read’s edges (<5 bp from read ends) were discarded. SNPs with low quality scores (<Q20) or low read coverage (<10x) were also eliminated. Furthermore, sequences with a length of 100 bp at both sides of the SNP in the reference genome were extracted and aligned with the assemblies to verify the SNPs using BLAT v34 [30]. If the length of the aligned region was shorter than 101 bp, the corresponding SNP was removed.

### Validation and functional analysis

To confirm the bioinformatics results, the regions containing the predicted InDels and SNPs were re-sequenced with traditional Sanger sequencing. The confirmed variants were further examined for their expression in *S*. *mutans* C180-2 and C180-2FR by quantitative PCR (qPCR).

For validation by Sanger sequencing, primers (see Supporting Information, S1 Table) targeting the predicted variants were designed based on the reference genome of *S*. *mutans* strain UA159 [31]. Proofreading PCR was carried out using the extracted genomic DNA of *S*. *mutans* C180-2 and C180-2FR as templates. The PCR products were purified with the MSB Vario Cleanup kit (Invitek, Berlin, Germany). Two purified PCR products per variant were sequenced at GATC Biotech (Konstanz, Germany). The nucleic acid sequence of each variant from C180-2 was aligned to the corresponding sequence from C180-2FR and the sequence from the reference strain *S*. *mutans* LJ23 using BioEdit software (Tom Hall, Ibis Bioscience, CA, USA).

To understand the potential roles of the confirmed variants in the *S*. *mutans* fluoride-resistant strain, expression of the selected genes was compared between C180-2 and C180-2FR using qPCR. Both strains were grown in BHI broth until early exponential phase (OD_600_ = 0.2), late exponential phase (OD_600_ = 0.8) and stationary phase (average OD_600_ = 1.1). Next, 6 ml of early exponential culture or 2 ml of late exponential or stationary culture was centrifuged at 16100 x g for 2 min at room temperature, and the pellets were stabilized with RNAprotect Bacteria Reagent (Qiagen, Hilden, Germany). Total RNA of the sample was extracted by beating with 0.1 mm glass beads followed by RNA purification using the Genejet RNA kit (Thermo Scientific, MA, USA). Genomic DNA contamination was removed with the TURBO DNA-*free* Kit (Life Technologies, Carlsbad, USA). Subsequently, cDNA was synthesized using a RevertAid First Strand cDNA Synthesis Kit (Thermo Scientific, MA, USA) with random hexamer primers. Expression of the selected genes was examined with specific PCR primers using SYBR Green based qPCR. Primer sequences and annealing temperatures are given in the Supporting Information (S2 Table). Specificity of the PCR reactions was confirmed by melting curve analysis. Expression levels were normalized using the expression of two housekeeping genes *recA* and *gyrA* [32,33]. Expression of different genes was expressed as 2^-(∆Ct)^, where ∆Ct = (Ct_gene of interest_—Ct_geomean of housekeeping genes_). Fold changes in gene expression were determined by 2^-(∆∆Ct)^, where ∆∆Ct = ∆Ct_C180-2FR_—average ∆Ct_C180-2_. The experiment was independently repeated three times.

### Statistical Analysis

The fold-change data were analyzed with GraphPad Prism (version 4.00, GraphPad Software, San Diego California, USA). Student’s *t* test was used to compare the expression of each selected gene in *S*. *mutans* C180-2 with that in C180-2FR for each growth phase. The data were log2 transformed before analysis. Because the expression of 9 genes was compared, the significance level (α) for Student’s *t* test was adjusted according to the Bonferroni correction. Differences were considered statistically significant at *p* < 0.005.

## Results and Discussion

### Characteristics of *S*. *mutans* C180-2 and C180-2FR

To confirm the fluoride resistance of *S*. *mutans* C180-2FR, both C180-2 and C180-2FR were plated on BHI agar plates containing either no fluoride or 80–500 ppm fluoride. C180-2FR was able to grow on all plates, including those containing 500 ppm fluoride. However, the colonies were much smaller on the plates containing more than 160 ppm fluoride compared to the plates without fluoride. No growth of C180-2 was observed on the plates containing more than 80 ppm fluoride. These results are in agreement with our previous study [17].

**Fig 1:**
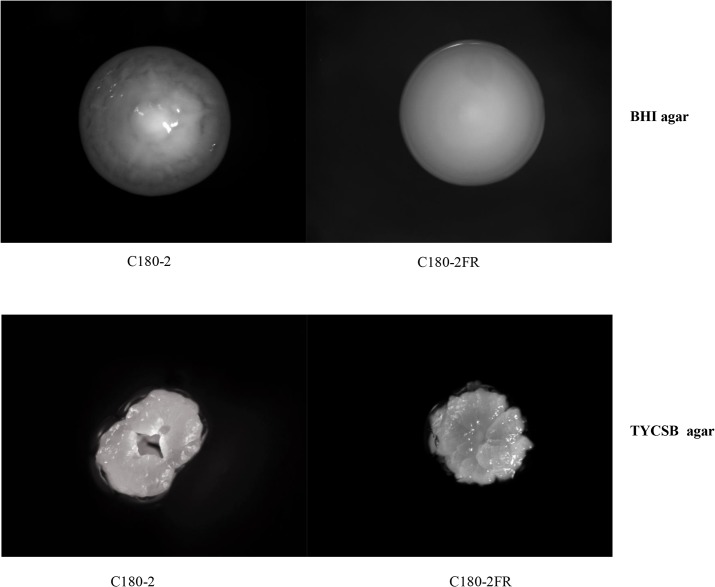
Colonies of *S*. *mutans* C180-2 and C180-2FR. *S*. *mutans* C180-2 and C180-2FR were grown anaerobically on BHI agar or TYCSB agar for 3 days. The images of the colonies on the agar plates were taken with an Anxion stereo-microscope with 50x magnification.

The growth rates (in BHI without fluoride) of these two strains also differ. The doubling time of C180-2FR was 74.5 ± 13.2 min, which is significantly longer than 50.9 ± 4.8 min of C180-2 (*p* < 0.05). Moreover, the colony morphology on BHI agar and TYCSB agar plates of the two strains showed clear differences (Fig 1). On BHI agar, the fluoride-resistant strain lost the textural structure of C180-2 and became flat. On TYCSB agar, the colony of the fluoride-resistant strain displayed a “frosted glass” appearance and was surrounded by a transparent extracellular polysaccharide layer, which was similar to that of C180-2. However, it lost the typical “bundt cake” shape of C180-2. To our knowledge, the differences in colony morphology have not been previously reported. The causes of these differences are not clear. It is hard to extrapolate whether the changes in colony morphology in C180-2FR were caused by fluoride or due to genome mutations unrelated to fluoride. Further research is needed to establish a causal relationship between a fluoride-resistant mutation and the changes in colony morphology.

### Comparison of genome sequences between *S*. *mutans* C180-2 and C180-2FR

Genomic DNA of the fluoride-sensitive (C180-2) and fluoride-resistant (C180-2FR) strains was sequenced with the Illumina GAIIx platform, which generated a large amount of paired-end reads. The total number of reads for each strain was comparable and had more than 200-fold coverage of the *S*. *mutans* genome. After quality filtering, 510 million reads remained for C180-2 and 350 million reads for C180-2FR. The genome assembly of C180-2 consisted of 28 scaffolds with a genome size of 2,051,830 bp and an average GC content of 36.68%. The assembly of C180-2FR was made up of 27 scaffolds with a genome size of 2,052,483 bp and an average GC content of 36.68%. These data indicated that the assembly of either strain was successful (see assembly statistics in Table 1).

**Table 1:** Assembly statistics of *S*. *mutans* C180-2 and C180-2FR.

	*S*. *mutans* C180-2		*S*. *mutans* C180-2 FR	
	Scaffold	Contig	Scaffold	Contig
**Total Number**	28	57	27	64
**Total genome size (bp)**	2,051,830	2,051,830	2,052,483	2,048,525
**N50 (bp)**	203,788	95,690	245,391	90,474
**Sequence GC (%)**	36.68	36.68	36.68	36.68

Three types of variant analyses, including structural variations, insertion-deletion variant (InDel) calling, and SNP calling, were carried out on the scaffolds of both strains. Typically, the purpose of structural variation analysis is to identify large deletions, insertions, duplications and translocations in the tested strains. This analysis has been used to identify the acquisition of virulence and antimicrobial resistance genes and to study the evolution of bacterial pathogens [34]. In our case, we expected high homology between the two tested strains because the fluoride-resistant C180-2FR strain was obtained by plating a high density of C180-2 cells on agar plates containing 500 ppm fluoride in the laboratory [7]. Large insertion/deletions in the genome are unlikely to occur during this process. The structural analysis confirmed the extremely high homology between the two strains. No genome rearrangement was detected (data not shown).

Using stringent filtering criteria (see Materials and Methods), we found 4 InDels in different coding regions in the two strains (Table 2). Unfortunately, we could not confirm any of these predictions using the conventional Sanger sequencing. At the predicted locations, the nucleic acid sequences in both strains were identical. The reasons for the failure in InDel prediction are unclear because stringent criteria were used in the analysis. Further studies are needed to improve this analysis.

**Table 2:** Identified InDels in the genome of *S*. *mutans* C180-2 and C180-2FR.

InDel_type*	InDels	C180-2 scaffold	C180-2FRscaffold
I3	GGG	scaffold16	scaffold12
D9	TTTTGGCTG	scaffold17	scaffold5
I1	A	scaffold15	scaffold11
I1	A	scaffold15	scaffold11

* InDel_type indicates the number of bases inserted (I) or deleted (D) in C180-2FR, when compared to C180-2.

**Fig 2:**
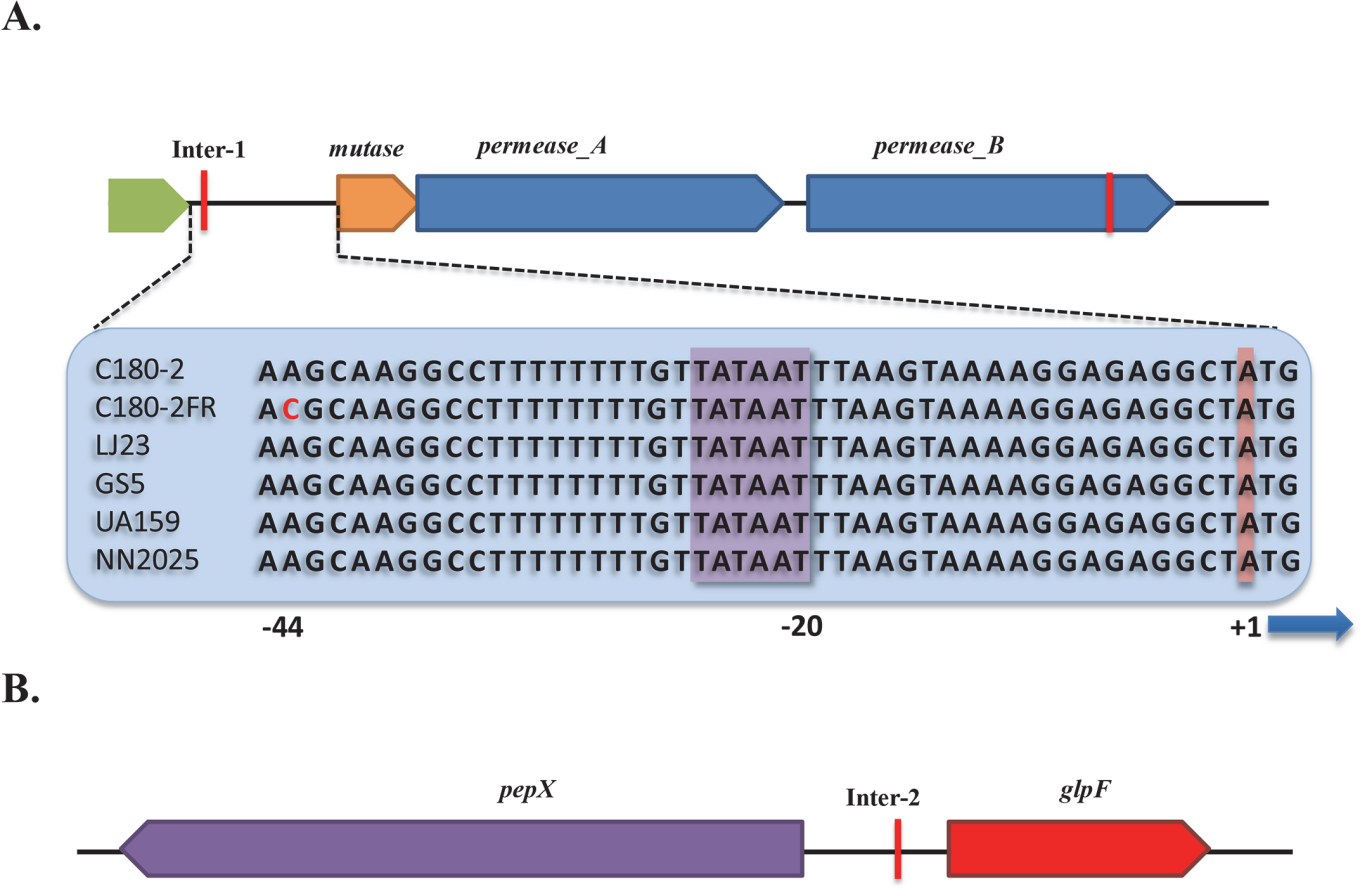
Gene organization at two intergenic regions in *S*. *mutans* C180-2 and C180-2FR. A. Orientation of the genes up- and down-stream of the intergenic region 1 (Inter-1). The sequences of Inter-1 in C180-2, C180-2FR, *S*. *mutans* UA159, *S*. *mutans* LJ23, *S*. *mutans* NN2025 and *S*. *mutans* GS5 are given in the blue bar. The red letter indicates the SNP; the purple box indicates the TATAAT box; the red box indicates the transcription start site of the operon; B. Orientation of the genes up- and down-stream of the intergenic region 2 (Inter-2). In both A and B, the red lines indicate the location of the SNPs.

Table 3 shows the results of the SNP analysis. All predicted SNPs were validated by Sanger sequencing. To exclude possible sequencing errors, we sequenced two PCR products per target region. For each SNP, both PCR products showed the same sequencing result. Moreover, the corresponding sequences of the reference strain LJ23 were also aligned to the sequences from C180-2 and C180-2FR. At the position of each SNP, the nucleotide in C180-2 was identical to that in LJ23. Thus, all SNPs predicted for C180-2FR were confirmed. Out of the 8 SNPs, 2 were located in intergenic regions, and 6 were non-synonymous coding substitutions that were located in 5 different Open Reading Frames (ORFs). These ORFs were further annotated. The pyruvate kinase (*pyk*) gene contains 2 SNPs. We also located the genes that are controlled by the promoters in the mutated intergenic regions. These genes include a putative mutase, a putative permease chloride channel (named *permease_A*), a putative glycerol uptake facilitator protein (*glpF*) and an X-prolyl dipeptidyl aminopeptidase (*pepX*). Fig 2 shows the organization of these gene clusters and their relation to the intergenic SNPs. Interestingly, the downstream *permease_A* gene is a paralog of another putative permease chloride channel (named *permease_B*), which contains a non-synonymous SNP. These related permeases, *permease_A* and *permease_B*, have 58% identical amino acids.

**Table 3:** Identified SNPs in the genome of *S*. *mutans* C180-2 and C180-2FR.

C180-2		C180-2FR		Mutation type	Gene
SNP base	Amino acid	SNP base	Amino acid		
C	R	T	C	nonsyn^a^	*smc*: putative chromosome segregation ATPase
G	T	A	I	nonsyn	*furR*: putative ferric uptake regulator protein
G	V	A	I	nonsyn	*permease_B*: putative permease chloride channel
A	M	G	V	nonsyn	*pyk*: pyruvate kinase
T	Y	G	D	nonsyn	*pyk*: pyruvate kinase
T	E	G	A	nonsyn	*holA*: DNA polymerase III subunit delta
A	—	C	—	intergenic^b^	—
C	—	A	—	intergenic^c^	—

a nonsyn: non-synonymous coding SNP.

b This intergenic region is located upstream of a putative mutase and a putative permease chloride channel (*permease_A*).

c This intergenic region is located between the *pepX* and *glpF* genes.

### Functional validation of SNPs by gene expression

**Fig 3:**
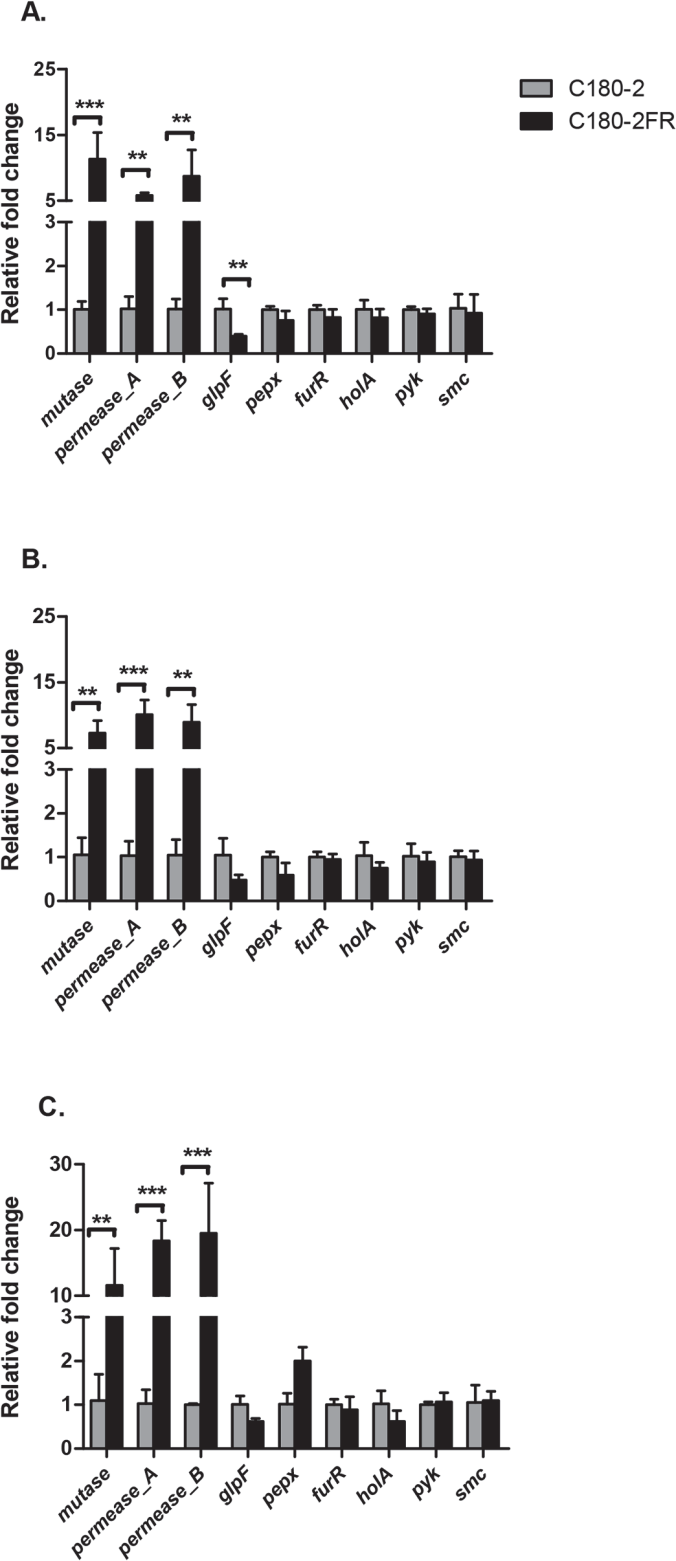
Comparison of gene expression between *S*. *mutans* C180-2 and C180-2FR. *S*. *mutans* C180-2 and C180-2FR at early exponential phase (A), late exponential phase (B) and stationary phase (C). Overall expression of each selected gene in C180-2FR relative to that in C180-2 is presented as average fold-change ± SD. This experiment was repeated 3 times. All tested genes are categorized into three groups based on the type of relative fold changes. The significance level (α) was set at 0.005 (after Bonferroni correction). ** indicates *p* < 0.005. *** indicates *p* < 0.0005.

To explore the influence of the identified SNPs on gene function, we further compared expression of the selected genes between C180-2 and C180-2FR at three growth phases. In total, the expression of 9 genes was examined, using *recA* and *gyrA* as the housekeeping genes. Fig 3 demonstrates the fold change in expression of the genes in C180-2FR relative to C180-2 at early exponential, late exponential and stationary phase. Irrespective of the growth phase, expression of three genes was approximately 10-fold higher in C180-2FR than in C180-2 (*p <* 0.005). These 3 genes were neighboring genes: two of them (*mutase* and *permease_A* genes) are in one operon under control of the mutated intergenic region (Inter-1); the other gene is a *permease_B* gene. Two genes (*glpF* and *pepX*) were downstream of the other mutated intergenic region (Inter-2). Expression of the *glpF* gene in C180-2FR was lower than in C180-2. However, this result was only significant in early exponential phase (p = 0.003). No differences in expression of the other tested genes were observed between C180-2 and C180-2FR.

It appeared that the genes that were differentially expressed in the fluoride-sensitive and fluoride-resistant strains were mostly related to the SNPs at the intergenic regions. At Inter-1, the nucleic acid residue at position -44 was changed from A (C180-2) to C (C180-2FR). The sequence of Inter-1 (see Fig 2) shows the TATAAT box, at position -20, and the point mutation 19 bp upstream of the TATAAT box, which is near the -35 element of the promoter. Because the binding site for RNA polymerase spans both -10 and -35 elements [35], this point mutation likely changed the affinity of RNA polymerase. The consistently high expression of *mutase* and *permease_A* in C180-2FR throughout bacterial growth strengthened the link between the SNP in Inter-1 and the high expression of this operon. However, we could not explain the high expression of the *permease_B* gene in C180-2FR because this gene has its own promoter region, in which there was no mutation identified (see Fig 2). We noticed that the *permease_B* gene contains a non-synonymous coding SNP, where a valine residue in C180-2 was replaced by an isoleucine residue in C180-2FR. Whether this SNP is related to the high *permease_B* gene expression needs further investigation. The Inter-2 region controls both the *pepX* and *glpF* genes (Fig 2). The SNP in this region seems to affect expression of the *glpF* gene in C180-2FR, though the effect was only significant in early exponential phase.

Interestingly, both *permease_A* and *permease_B* gene were found to be homologs of *eriC* in *Pseudomonas syringae*. Recent studies showed that *eriC* is a fluoride antiporter, instead of a chloride transporter as indicated by its gene name [36]. This transporter protein greatly prefers F^-^ over Cl^-^ and exchanges F^-^ for H^+^ with 1-to-1 stoichiometry [37]. Our finding indicated that the *S*. *mutans* fluoride resistant strain is likely to be protected from high fluoride concentrations in the environment through high expression of *permease_A* and *permease_B*. These two proteins could act as H^+^-coupled antiporters by exporting F^-^ to below its extracellular level. It is worth mentioning that, in the current study, the bacterial cells grew in fluoride-free media. Thus, high expression of the fluoride antiporters in C180-2FR was not induced by fluoride but rather an endogenous expression.

There is no clear link between the function of *glpF* and the fluoride resistance of *S*. *mutans*. *glpF* codes for a putative glycerol uptake facilitator protein [38]. It functions as a membrane channel that selectively transports water, small neutral molecules and ions out of and between cells. In *Escherichia coli*, it selectively mediates the diffusion of glycerol into the cytoplasm. *E*. *coli* uses glycerol as a carbon source for glycolysis and for lipid biogenesis [39]. In *Lactococcus lactis*, GlpF can transport both glycerol and water and mediates the growth of bacterial cells in the presence of glycerol [40]. The function of *glpF* is likely to be related to bacterial cell growth. The lower expression of this gene in the fluoride-resistant strain, as observed in this study, may be related to the slow growth rate of the strain rather than to the fluoride resistance of the strain.

Another interesting candidate gene listed in Table 3 is pyruvate kinase. This gene contains two SNPs, and it is an important glycolytic enzyme in the glycolysis pathway. Previous studies have shown that fluoride affects bacterial metabolism in two ways: by direct inhibition of cellular enzymes (directly or in combination with metals) or by enhancing proton permeability of cell membranes in the form of hydrogen fluoride (HF) [41]. One of the enzymes that are directly inhibited by fluoride is thought to be enolase (as mentioned above). In glycolysis, enolase catalyzes the conversion of 2-phosphoglycerate to phosphoenolpyruvate (PEP), while pyruvate kinase converts PEP to pyruvate. Although fluoride inhibition of enolase has been shown in many *in vitro* studies, it was still in question whether this inhibition would occur in intact bacterial cells because only purified or extracted enolase was examined in those studies [42]. As for fluoride inhibition of pyruvate kinase, there has been only one report so far [43]. In that study, the fluoride inhibition on pyruvate kinase was considered unlikely to occur in bacterial cells because the inhibitory fluoride concentrations for this enzyme were approximately 10 to 100 times higher than those required for enolase inhibition [41]. In our functional test (qPCR), we did not find any difference in pyruvate kinase expression between C180-2 and C180-2FR (Fig 3). Nevertheless, given the number of SNPs found in this enzyme, the central role of this enzyme in glycolysis, and the characteristic inhibition of fluoride on bacterial glycolysis, the potential role of pyruvate kinase in bacterial fluoride resistance merits further study.

In summary, by using whole-genome shotgun sequencing followed by genome comparisons, we were able to discover and validate 8 SNPs in the genome of the fluoride-resistant *S*. *mutans* strain. This study provided solid support to the previous assumption that stable genotypic changes exist in the fluoride-resistant strain. The identified genes/regions provide new targets for studying the molecular mechanisms of microbial fluoride resistance. Moreover, the initial functional analysis of these genes revealed that two of the SNPs were related to a recently discovered fluoride antiporter and that the corresponding genes were highly expressed in the fluoride-resistant *S*. *mutans* strain throughout growth of the strains and independent of the presence of fluoride. Although no direct link between these two SNPs and fluoride resistance was established, our results indicated a potential role of this antiporter in microbial fluoride resistance.

## Supporting Information

S1 Table Primers for Sanger sequencing. (DOCX)

S2 Table qPCR primer sequences and annealing temperatures. (DOCX)
