# Supplementary material for: Identification and Functional Analysis of Genome Mutations in a Fluoride-Resistant Streptococcus mutans Strain
Source: PLoS One. 2015 Apr 9;10(4):e0122630. doi: 10.1371/journal.pone.0122630 (PMC4391945; doi:10.1371/journal.pone.0122630)
Supplement: S1 Table — (DOCX) [file pone.0122630.s001.docx]

**S1 Table.** Primers for Sanger sequencing

| Gene name | Primer | Sequence (5'-3') |
| --- | --- | --- |
| *smc* | Forward | AATAACTTGGCTGAAGATTA |
|  | Reverse | TTAAGATATCTTTTCGACTTCT |
| *furR* | Forward | TCCACATATGGGACTGCATTCTCATAATAA |
|  | Reverse | ACTGGAATTCTTAATCGGCTTTTTTAGTTTTCTTG |
| *permease_B* | Forward | GAATTCATTAAGCAGTAACCTTTTGTGCACCATAA |
|  | Reverse | CATATGGGGATTAAGATCAAGTCTTTT |
| *pyk* | Forward | GCCACATATGAATAAACGCGTAAAAATTGT |
|  | Reverse | CATGCCGCGGTTATTGAACGGTACGAACAC |
| *holA* | Forward | GCCACATATGATTGCTATAGAAATGATTGAAAAGTTAAA |
|  | Reverse | ACTGGAATTCTCATCCCCGATTTTCATGAGCTAATTT |
| *Inter-1* | Forward | CATATGCTTAATCCCCATCTAATGCT |
|  | Reverse | GCATGCACTGATATTACTGGCTATTTA |
| *Inter-2* | Forward | TAACATCCATTTGTCTGTTAT |
|  | Reverse | GCATGCCCGATATAACTGTATTGATT |
