# Supplementary material for: Identification and Functional Analysis of Genome Mutations in a Fluoride-Resistant Streptococcus mutans Strain
Source: PLoS One. 2015 Apr 9;10(4):e0122630. doi: 10.1371/journal.pone.0122630 (PMC4391945; doi:10.1371/journal.pone.0122630)
Supplement: S2 Table — (DOCX) [file pone.0122630.s002.docx]

**S2 Table.** qPCR primer sequences and annealing temperatures

| Gene name | Primer | Sequence(5'-3') | Product  length  (bp) | Annealing  temperature  (°C) |
| --- | --- | --- | --- | --- |
| *smc* | Forward | CAATCTTATGTCTGGTGGTGAA | 106 | 57 |
|  | Reverse | AGCCTCAACCTCATCCAA |  |  |
| *furR* | Forward | TCCTCAGTATCCAAGTATG | 162 | 59 |
|  | Reverse | TTACCACAAGACTCACAA |  |  |
| *permease_B* | Forward | AGATGCTAATCCTTGGTA | 140 | 59 |
|  | Reverse | TATGGTCTTCCTCTTCAA |  |  |
| *pyk* | Forward | GGTGAAGATGGCTATTGG | 85 | 59 |
|  | Reverse | CATTGGCTCCTTCTGTAAT |  |  |
| *holA* | Forward | GGAGAAGATGAGATTAAGT | 187 | 57 |
|  | Reverse | AAGTGAGTGTTCTTGAAT |  |  |
| *mutase* | Forward | ATGGTGGAGCGATATGTA | 146 | 59 |
|  | Reverse | TGTTTAGAAAGACGAATGACT |  |  |
| *permease_A* | Forward | TTACTGCTGCTGGTATGG | 131 | 57 |
|  | Reverse | TGCTGATAAGGTTAATACTGTTAG |  |  |
| *glpF* | Forward | GTTACCAGATACATTACCA | 154 | 59 |
|  | Reverse | TACTGCTCTACTCGTTAT |  |  |
| *pepX* | Forward | TATGGCTGACTGGACTAA | 115 | 57 |
|  | Reverse | TTCCGCAATAATGACCTTA |  |  |
